# A qualitative study of organisational response to national quality standards for 7-day services in English hospitals

**DOI:** 10.1186/s12913-021-06213-w

**Published:** 2021-03-06

**Authors:** Elizabeth Sutton, Julian Bion, Russell Mannion, Janet Willars, Elizabeth Shaw, Carolyn Tarrant

**Affiliations:** 1 Department of Health Sciences, University of Leicester, Leicester, UK; 2 Intensive Care Medicine, University Department of Anaesthesia & Critical Care, University of Birmingham, Birmingham, UK; 3 Health Services Management Centre, University of Birmingham, Birmingham, UK

**Keywords:** Organisational culture, Healthcare quality improvement, Qualitative research

## Abstract

**Background:**

National standards are commonly used as an improvement strategy in healthcare, but organisations may respond in diverse and sometimes negative ways to external quality demands. This paper describes how a sample of NHS hospital trusts in England responded to the introduction of national standards for 7-day services (7DS), from an organisational behaviour perspective.

**Methods:**

We conducted 43 semi-structured interviews with executive/director level and clinical staff, in eight NHS trusts that varied in size, location, and levels of specialist staffing at weekends. We explored approaches to implementing standards locally, and the impact of organisational culture and local context on organisational response.

**Results:**

Senior staff in the majority of trusts described a focus on hitting targets and achieving compliance with the standards. Compliance-based responses were associated with a hierarchical organisational culture and focus on external performance. In a minority of trusts senior staff described mobilising commitment-based strategies. In these trusts senior staff reframed the external standards in terms of organisational values, and used co-operative strategies for achieving change. Trusts that took a commitment-based approach tended to be described as having a developmental organisational culture and a history of higher performance across the board. Audit data on 7DS showed improvement against standards for most trusts, but commitment-focused trusts were less likely to demonstrate improvements on the 7DS audit. The ability of trusts to respond to external standards was limited when they were under pressure due to a history of overall poor performance or resource limitations.

**Conclusions:**

National standards and audit for service-level improvement generate different types of response in different local settings. Approaches to driving improvement nationally need to be accompanied by resources and tailored support for improvement, taking into account local context and organisational culture.

**Supplementary Information:**

The online version contains supplementary material available at 10.1186/s12913-021-06213-w.

## Background

Changes in the organisation and delivery of healthcare are often driven through national quality standards, monitoring of attainment of targets, financial incentives, and inspections. External demands for quality help focus management attention, but can generate negative organisational responses, including tunnel vision and gaming [1]. Some organisations will respond more positively to external demands for quality than others. Drawing on institutional theory, Oliver (1991) maps out different strategic responses enacted by organisations in response to external pressures for conformity - from acquiescence and active compromise with partial compliance, through to more negative responses including avoidance, defiance and manipulation. Oliver argues that organisational response will be influenced by local context including resources, power and choice constraints, and motives of maintaining stability and legitimacy [2].

In their study of organisational responses to external demands for finance and quality, Burnett et al. [3] found that positive responses were fostered by perceived coherence of the external demands, managerial competence to align demands with an overall quality improvement strategy, and managerial stability. When these factors were absent, organisations were more likely to respond with habitual or symbolic compliance decoupled from improvement efforts.

Organisational responses to external demands for quality are also likely to be shaped by organisational culture [4]. Organisational culture describes the beliefs, values, attitudes and behavioural norms within an organisation as well as its routines and traditions. It incorporates the way that people understand and make sense of practices, and what is seen as legitimate and acceptable within any given organisation [5]. Evidence suggests that high-performing organisations have distinct cultural features: positive norms and values, strong feelings of belonging, trust and cohesion; an ‘outward facing’ orientation, and flexibility to embrace change [6, 7]. In contrast, organisations with below-average performance on patient outcomes and quality measures are more likely to exhibit a non-collaborative, hierarchical culture and a lack of cohesive mission and vision, and dysfunctional external relationships [7].

The ability of an organisation to change and improve in response to external standards may be limited by resources and capacity. Organisations identified as ‘struggling’ in terms of quality and outcome measures are more likely to have experienced problems with resources and inadequate infrastructure, faced system shocks such as senior leadership turnover, or experienced financial failure [7] Making change happen is difficult when healthcare organisations lack organisational ‘slack’ – the time and resources to enable learning and creativity [8].

Overall, organisational response to external demands for service level change are likely to be shaped by an interplay between leadership [9] organisational context and culture, and the extent of strain on the organisation.

We investigated how organisations responded to national quality standards, and the factors that shaped organisational response, in a sample of NHS hospital trusts in England (organisations that manage and deliver hospital services) during the introduction of the national policy of 7-day service standards (7DS). The 7DS policy was formulated in 2013 and launched in 2014/15, partly in response to concerns about increased patient mortality following emergency admission to hospitals at weekends [10]. The policy took the form of ten clinical standards (Table 1), with four identified as urgent priorities for improving patient outcomes: time to first consultant review; access to consultant-directed testing; access to consultant directed interventions; and ongoing consultant review of patients’ high dependency needs [11]. The extent to which trusts met 7DS is measured by internal performance audit against a standardised framework, with benchmarked publicly reported results [11]. Implementing 7DS required significant modifications to structures and processes of care with resource implications such as altered staffing patterns and greater consultant involvement in front-line care at weekends. This challenged organisations and clinicans to develop new ways of working. While the principle of enhanced consultant involvement in front-line patient care had strong professional support [12], the rationale was contested [13] as was its affordability [14]. Some Trusts had more resources and flexibility of staffing and considered that they were already providing substantial consultant input at weekends, whereas others had fewer resources and were already stretched in terms of matching staffing to patient admissions.

**Table 1:** Seven Day Services Clinical Standards (NHS England, September 2017)

1	**Patient Experience**: Patients, and where appropriate families and carers, must be actively involved in shared decision making and supported by clear information from health and social care professionals to make fully informed choices about investigations, treatment and on-going care that reflect what is important to them. This should happen consistently, seven days a week.
2^*^	**Time to First Consultant Review**: All emergency admissions must be seen and have a thorough clinical assessment by a suitable consultant as soon as possible but at the latest within 14 h from the time of admission to hospital.
3	**Multidisciplinary Team Review**: All emergency inpatients must be assessed for complex or on-going needs within 14 h by a multi-professional team, overseen by a competent decision-maker, unless deemed unnecessary by the responsible consultant. An integrated management plan with estimated discharge date and physiological and functional criteria for discharge must be in place along with completed medicines reconciliation within 24 h.
4	**Shift Handovers**: Handovers must be led by a competent senior decision maker and take place at a designated time and place, with multi-professional participation from the relevant in-coming and out-going shifts. Handover processes, including communication and documentation, must be reflected in hospital policy and standardised across seven days of the week.
5^*^	**Diagnostics**: Hospital inpatients must have scheduled seven-day access to diagnostic services, typically ultrasound, computerised tomography (CT), magnetic resonance imaging (MRI), echocardiography, endoscopy, and microbiology. Consultant-directed diagnostic tests and completed reporting will be available seven days a week: • Within 1 h for critical patients • Within 12 h for urgent patients • Within 24 h for non-urgent patients
6^*^	**Intervention / key services**: Hospital inpatients must have timely 24 h access, seven days a week, to key consultant-directed interventions that meet the relevant specialty guidelines, either on-site or through formally agreed networked arrangements with clear written protocols.
7	**Mental Health**: Liaison mental health services should be available to respond to referrals and provide urgent and emergency mental health care in acute hospitals with 24/7 Emergency Departments 24 h a day, 7 days a week.
8^*^	**Ongoing Review**: All patients with high dependency needs should be seen and reviewed by a consultant TWICE DAILY (including all acutely ill patients directly transferred and others who deteriorate). Once a clear pathway of care has been established, patients should be reviewed by a consultant at least ONCE EVERY 24 HOURS, seven days a week, unless it has been determined that this would not affect the patient’s care pathway.
9	**Transfer to community, primary and social care**: Support services, both in the hospital and in primary, community and mental health settings must be available seven days a week to ensure that the next steps in the patient’s care pathway, as determined by the daily consultant-led review, can be taken.
10	**Quality Improvement**: All those involved in the delivery of acute care must participate in the review of patient outcomes to drive care quality improvement. The duties, working hours and supervision of trainees in all healthcare professions must be consistent with the delivery of high-quality, safe patient care, seven days a week.

^*^Priority standard (2, 5, 6 & 8)

The implementation of national 7DS in the NHS in England therefore provides a valuable case study through which to study organisational responses to external demands for quality implemented through national standards and monitoring. We aimed to explore patterns in how organisations responded to the four priority standards and targets specifically from an organisational culture and behaviour perspective.

## Methods

The study is part of the High-intensity Specialist Led Acute Care (HiSLAC) project, which evaluated weekend care for acute medical patients, with a particular focus on care quality and specialist (consultant) staffing [15] across twenty NHS hospital Trusts in England. We selected eight of these Trusts for an in-depth study of the factors influencing the response to the four prioritised 7DS standards. The trusts are labelled in this paper by their original site identifier resulting in identifiers that do not run consecutively from 1 to 8. Trusts were selected to ensure representation of diversity in weekend specialist intensity [16], size and location.

To assess trust achievement against the four prioritised national standards for 7DS we compiled trust level data from the national audits of 7DS [11], (see Table 2). To study organisational responses, characterise local context, and explore staff views of how organisational culture shaped response to the standards, we conducted semi-structured interviews with members of the senior management team and frontline staff in each trust. We also accessed national reports on care quality and financial performance for each trust (Care Quality Commission reports), to enable us to characterise participating trusts based on external indicators of quality and financial strain.

**Table 2:**
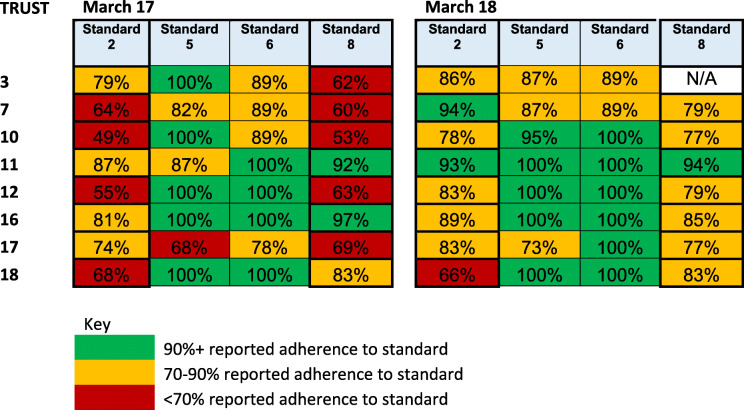
Performance against four quality standards at weekends, from national audit data, March 2017, March 2018

We purposively sampled five to six members of staff in each trust to participate in semi-structured interviews. Staff were sampled to cover a range of roles including board members, and frontline clinical and non-clinical staff involved in implementation of 7DS. Participants were approached by e-mail via a project lead in each site. A piloted interview topic guide (Additional file 1) used the Competing Values Framework (CVF), a validated model of organisational culture [5, 17], to stimulate discussion and reflection. The CVF uses two main dimensions; the first describes how internal processes are structured within the organisation and the second describes the orientation of the organisation to the outside world. This gives rise to four distinct cultural ‘types’: Clan, Developmental, Hierarchical and Rational. Organisations are not simply categorised as one or other of these four types, but may have values reflective of more than one type, or may have a stronger pull to one particular quadrant. Interviewees were asked to identify the cultural type(s) from the CVF that best described the overall culture in their organisation in relation to implementing change. They were asked to discuss their organisational culture and local context, how they had approached implementing the priority standards, and the factors impacting on their response.

Interviews were conducted by ES and JW between November 2017 and March 2018. Interviews took place in offices at the hospital either face-to-face, or by telephone for the convenience of the interviewee. The researchers had no previous relationship with the participants. Interviews lasted between 30 min and an hour and were audio-recorded. Recordings were transcribed, anonymised, and imported into Nvivo 11 software. ES and ESh coded and analysed the data using a thematic analysis approach [18], in collaboration with CT. A selection of transcripts were open-coded, then a full coding frame was developed, incorporating open codes and themes related to the CVF, and was used to code the full data set. Themes and codes were reviewed and discussed within the team until no new themes emerged (See Additional file 2). We used case summaries and cross-case narratives to interpret findings.

## Results

Compliation of national audit data on the four prioritised national standards for 7DS [11] demonstrated variation between trusts in their achievement of the standards. (Table 2).

We conducted a total of 43 semi-structured interviews with staff who had knowledge of local implementation of 7DS. They included board level staff (medical directors, financial directors and chief medical officers), acute medical consultants, acute consultant rota co-ordinators, and senior nurses (for further information see Additional file 3). Quotes are labelled by Trust and role; our focus here is on organisational implementatation of change, meaning that we draw more heavily on the accounts of senior leaders than frontline staff in our findings.

We distinguished between two types of organisational response to the national 7DS: a compliance-focused response which centred on accountability and demonstrating adherence to external standards, and a commitment-focused response that involved aligning external standards with organisational goals and values to achieve change [19]. We describe how these orientations were expressed in approaches to implementation, and identify key features of the local context that shaped the way organisations responded to the standards. Trusts’ features and approaches are summarised in Table 3.

**Table 3:** Trust, context and culture

TRUST	NOTABLE FEATURES	ORGANISATIONAL CULTURE	FINANCIAL POSITION 2017–18	CQC QUALITY 2017–18	ORGANISATIONAL STRATEGY
03	District general hospital in an urban location. 50% shortfall in consultant staffing in A&E and reliance on bank/agency staffing in AMU in particular. Well networked to other services in the locality.	Hierarchical	Reduced financial deficit but still had large deficit of over £20 million	Requires Improvement	This organisation focused on complying with the standards. Policies, processes and protocols were important in this hospital. They had implemented some changes by the introduction of more acute care physicians but there was the sense that more consultants were needed in order to deliver.
07	Teaching hospital in an urban location in a deprived area. History of organisational turbulence as two separate organisations merged into one Trust a few years prior to fieldwork. Staff recruitment an issue in this Trust.	Hierarchical / clan	Agreed to deliver a deficit of no more than £35 million in 2018/19	Requires Improvement	Focus on compliance with standards. A merger between two different hospitals had led to a more hierarchical approach in order to effect change. Over the past few years they had focused on recruiting new staff for the emergency pathway but were still exploring how staff could work differently.
10	One hospital in a group of 3 in a relatively affluent urban area. Good links with other services in the locality. Recent change of board leadership; consultant body described as ‘the old fashioned firm structure’.	Hierarchical / clan	Had met their control total	Good	Focus on compliance with standards, use of audit to monitor. The hospital was reported to be hierarchical and reactive in implementing new policies and structures. The consultant body was reported to be ‘clannish’. Management were still working out where the gaps were in 7 day service targets. Changes to the consultant contract meant consultants were asked to conduct ward rounds at the weekend.
11	District hospital. Staff recruitment difficult. The location was felt to be a deterrent as it was an expensive area to live in. Trust was in financial special measures at the start of the fieldwork.	Hierarchical / clan	£12 million deficit against a planned deficit of £7 million	Requires improvement	Commitment-based strategies. Organisation with a community feel, with stability and loyalty. Focus was on best interests of patients. Delivery was through incremental change and collaboration.
12	Large teaching hospital and major trauma centre. Difficulties in recruiting to acute general medicine but not to specialist branches.	Hierarchical / clan	Deficit of over £8 million	Good	Focus on compliance with standards. There was seen to be a need for policies and procedures to maintain standards, and that innovations could be slow to implement and the organisation could suffer from micromanagement. New doctors were being told they would undertake acute medicine for 50% of the time and take part in the weekend rota, and contracts were short term.
16	One of three hospitals supplying acute and maternity services located in a deprived area. Good links and access to the services supplied by the other Trusts.	Developmental	In surplus by over £7 million	Good	Commitment-based strategies. The focus was seen as being on the best interests of patients. This site used cooperative and collaborative strategies including peer pressure to achieve change. Management was heavily invested in achieving consensual change.
17	Hospital in a rural location. Significant problems with staffing at all levels and a high number of locum consultants; great difficulties recruiting staff. A&E in nearby location closed in the evenings adding to pressure on acute services. Isolated from other services.	Hierarchical / clan	Financial special measures	Requires Improvement	Focus was on compliance with standards. The organisation was dealing with a legacy of poor performance and trying to instil good practice. There was a tension between encouraging innovation and controlling policies and procedures. The management did not have a specific plan or programme to meet the 7 day standards but were trying to use resources efficiently and centralise. They were working hard to recruit internationally, and improve the workforce with leadership programmes and centralise job plans. Yet there was a sense of great strain in this organisation.
18	Large teaching hospital. Interviewees state that they have been meeting 7 day standards for some time. The hospital is well staffed.	Developmental / rational	In surplus by over £20 million	Good	Commitment-based strategies. This site framed changes in terms of best interests of patients. Change was being achieved through collaborative strategies including gaining consensus through participatory discussions with staff.

## Compliance oriented responses

The majority of Trusts responded as might be expected, with senior leaders focused on hitting the targets laid out in the 7-day framework. We categorised five of the eight trusts as taking a predominately compliance-based approach (trusts 3, 7, 10, 12, and 17) (Table 3). Staff in these trusts described an emphasis on complying with meeting the standards.

*There was a big piece of work about where everyone was against the standards as a baseline, and then, what do you need to do to get yourself up to complying with standards […]. What we’ve been monitoring since then […] is compliance with that seven day standard (Trust 03, board member).*

Senior staff in these trusts were concerned about how they would be perceived externally, and had concerns about the potential consequences of failing to perform against these national standards.

Senior leaders tended to describe using command and control approaches to deliver against the standards, with little flexibility in how changes were to happen. In practice this meant a more top-down approach to implementing change, for example central coordination of changes to consultant rotas or the introduction of new roles for staff. Board members in three trusts (3, 10 and 12) described themselves as being innovative and open to bottom-up improvement, but argued that central control and direction was crucial in managing and implementing large scale change. In trust 10, for example, board members described being keen to support empowering staff to make improvements, but this was limited to addressing the ‘low hanging fruit’ of quality improvement rather than dealing with national directives.

*So we want to empower people to, to develop their, what they are doing, improve […] you know, not the big systematic problems but the things that just need [fixing] (Trust 10, board member).*

Top-down approaches, with formal rules and procedures, were seen as enabling control over the process of large scale change, and particularly important when the stakes were high – as in the case of 7DS where the Trust was under scrutiny.

*You’ve got to have some control in place to be able to see how you’re doing against [standards]* […] *(Trust 3, board member)*

There was evidence that top down approaches could drive changes forward, and the majority of trusts that leaned towards a compliance-based approach showed an improvement on the priority 7DS in terms of the national 7DS audit (Table 1). When changes were imposed, however, there was evidence that consensus and commitment among frontline staff was lacking, with the legitimacy of the standards being questioned.

*I’m not sure that all these patients do need the reviews that are being asked for […] That’s the argument I’m getting from [staff] (Trust 7, board member)*.

There was also seen to be a lack of a sense of collective responsibility among the workforce around weekend working. In Trust 10, for example, there was concern expressed that senior clinical staff, being directed to take undesirable shifts, were simply passing these on to more junior consultants who found it difficult to refuse.

*It is a frequent occurrence for senior consultants to ring junior consultants to say ‘can you do my […] on call for me I’ve got x or y, or I just can’t do it’. […] So it’s not ‘you scratch my back and I’ll scratch yours’, it’s ‘you’re junior you’ll do it’. So that is not good for morale.* *(Trust 10, Consultant)*.

There was little sense in these trusts, at board or frontline level, of engagement with the standards as a lever to make genuine improvements to the quality of service delivery. As a result the implementation of the standards was arguably disconnected from longer term goals around improving quality.

*And at the moment, unfortunately because of the way the pressures that we are all under collectively [… ] we end up in a system which is more controlling. […] It means that actually we spend our time thinking about the next month or two rather than planning for 5 years. It is not sustainable (Trust 10, board member)*.

## Commitment oriented responses

Despite the standards being mandatory, three Trusts in our sample avoided a predominantly compliance-based response (trusts 11, 16 & 18). While senior leaders recognised the need to account for their performance, their concern was not primarily about outward displays of compliance and accountability. In these organisations, senior leaders were able to find ways to align external demands with the values of their organisation and frame them in terms of their overall goals of providing high quality patient care; this helped reconcile any issues about perceived legitimacy of the standards.

*So if you look at our values as an organisation so we did a piece of work on our values and expected behaviours. […] Our values as an organisation which we’ve all signed up to are patient centred and safe, friendly, professional and responsive, and […] you can definitely link wanting to improve services for patients at weekends to all of those values (Trust 11, board member)*.

These trusts were more likely to describe using collaborative and flexible approaches to implementation as opposed to imposing change. They were creative in their approaches to solving the problem of staffing the extended weekend service, reflecting their commitment to the spirit of the standards – providing better quality of care for patients at weekends – as opposed to demonstrating compliance with the letter of the standards. There was a concerted effort to engage frontline staff in the enterprise of improvement, and to involve them in decision-making, encouraging a perception of unity and willingness to work together to achieve weekend working.

*You just need to be mindful of the way you're doing it, when you try and change the system to allow specialists to work at the weekends. So you need to sit down with the specialists and engage with them. […] While it might take time, as I say, when you're doing it, you'll get a more positive outcome* *(Trust 18, Consultant)*.

Trust 16 also described using social strategies such as peer pressure to encourage recalcitrant staff to take on new ways of working, rather than imposing diktats from above.

*We had a meeting of all the medical specialties where we got the [clinical directors] to talk about their 7 day service implementation. […] The way it was presented was a very proud [clinical director] saying ‘look what we’ve done’. […] And within the space of about an hour those that had any ambivalence about it had actually changed their mind about it* *(Trust 16, board member)*.

Of fundamental importance to progressing change was a show of investment and reciprocal commitment from the Trust board towards consultants who were being asked to change the way they worked. In Trust 16, part of the implementation of 7DS was a trust-wide commitment to guaranteeing staff time off in lieu in the week to compensate for weekend working.

The scoring of these three trusts on the national audit was varied (Table 2). Trust 11 demonstrated improvements against the standards over the time period; for trusts 16 and 18 there was clear evidence from the qualitative research that they had engaged positively and creatively with the standards and were working to achieve genuine improvements, but this was not reflected in their data on the national audit. Trust 18’s performance against the standard of time to first consultant review remained low.

## Commitment or compliance orientation: the impact of organisational culture, context and performance

How trusts responded to the 7DS was shaped by organisational culture and local context (Table 3).

Staff described how both the orientation towards external standards, and the approach taken in implementing them, reflected the prevailing organisational culture. In trusts that took a compliance-based approach, interviewees identified the organisational culture as primarily hierarchical (characterised by top-down leadership approaches and structured around policies and rules), in some cases with features of clan culture (bonded by loyalty and emphasising tradition). Staff were used to responding to directives ‘from the top’, and felt they had little involvement or empowerment to shape organisational change.

*You’re just waiting, so some person in a [senior] position to then say ‘OK, we want to do that’ and then you work towards that. So obviously that is a consequence of having this kind of culture (Trust 12, Consultant).*

In trust 7, organisational instability played into the approach to implementing standards, and was argued to have necessitated a strongly ‘top-down’ approach to change in order to ensure compliance with the standards. There had been a recent merger between two different hospitals, and there was strong resistance to changing ways of working in one of these hospitals which was considered more ‘clan’ like and community-focused than the other. The resistance to changing their ways of working was such that it required a senior executive to mandate that the change should occur.

*In one of the areas […] they all said ‘No, we’re not doing it that way and we want to do it our way’. It required me to go in and say to them why they couldn’t do what they wanted to do *(Trust 7, board member).

In contrast, in two of the trusts which took a commitment-based approach (16 & 18), organisational culture was identified by senior leaders and frontline staff predominantly as developmental. In these trusts, leaders characterised their organisations as innovative, creative, and adaptive. They described how their prevailing culture enabled them to reframe the standards in relation to their own priorities, and to resist being tied to external judgements of quality.

*I think the way that we approached it was to completely ignore the political rhetoric […] I think there is something about a kind of culture in people taking on what they see to be a good thing, participating in that (Trust 16, Board member)*.

*I think they definitely have that mentality, that you know, we're different, or we're better, and we're not just going to do it […], because [other hospital] says, or NHS says, or whatever (Trust 18, Consultant)*.

One trust that demonstrated a predominantly commitment-focused approach appeared to be an exception in terms of how staff they described their organisational culture and its relationship to implementing 7DS. Trust 11 senior leaders emphasised their shared vision of improving patient care and their organisational values. However, unlike the other Trusts with a commitment orientation, frontline staff in this organisation identified the organisational culture as hierarchical and clan-like (Table 3). This discrepancy can be explained in relation to the Trust’s location, size and nature of its workforce. Trust 11 was a small district hospital with long-serving, loyal staff. Shared, cohesive values of frontline staff, along with a stable senior leadership team, meant it was easier for senior leaders to obtain endorsement from front line staff in order to implement the required changes.

*I think [the organisation has] got a good attitude to change. And I think most people who work here have. We - Sometimes change is forced upon us. But we are flexible enough that we will try and make it work for us, and we'll try very hard to make it work for us (Trust 11, Consultant)*.

Response to 7DS was also strongly shaped by the trusts’ overall history of performance against quality standards, their financial position, and the resource limitations within their local context. Notably, trusts 16 and 18, which described resisting a compliance-based approach, were operating at a surplus and were rated as ‘Good’ in the most recent Care Quality Commission inspection (Table 3). As such the senior leaders in these trusts did not feel under scrutiny or pressure to demonstrate compliance with external targets.

*The questions we ask ourselves […] would the patient have got better care if they were seen over the weekend or out of hours, or whatever you want to define as seven day service. […] We fill the framework [for 7DS reporting] when we have to, and ignore it if we can get away with ignoring it (Trust 18, board member)*.

In contrast, in those Trusts that were already dealing with a legacy of poor performance, senior leaders felt pressure to deliver against 7DS standards, but struggled to engage meaningfully in efforts to improve weekend working as they were already under strain from being placed in special measures. This drove a compliance-based approach to meeting targets, with some leaders acknowledging that directing resources towards the 7-day services agenda was not a priority in the face of other more pressing quality issues.

*We, tend to be focussed on results, delivering these results […] But seven day working [..] t's been subsumed, whilst - you're under more immediate actions to resolve those issues that you're addressing [being in special measures]. (Trust 17, board member)*.

For Trust 17, the difficulties faced by being in special measures were compounded by their isolation from other providers, which made it difficult to collaborate with others to deliver improved weekend services, alongside their longstanding difficulties in recruiting and retaining staff.

*I get the impression that it is not so much working towards a real 7 day service as much as it is an effort to keep patients safe with the bare minimum staffing levels because as I am sure you are aware staffing recruitment, retention is a huge issue with the Trust […] it is unrealistic to [..] expect a genuine 7 day service (Trust 17, Consultant).*

## Discussion

Our study highlights that a national, service-level, improvement initiative delivered through standards, targets and performance management, generated differential responses across organisations. We distinguished between commitment-based and compliance based responses [19]. Commitment-based approaches involved aligning standards with organisational goals, and generating consensus to drive change, along with the use of creative and flexible solutions. Trusts that orientated towards commitment-based approaches were more likely to be described by staff as having a predominantly developmental organisational culture. Senior leaders in these trusts facilitated a shared sense of purpose in improving the quality of care for patients around the clock, and resisted focusing on external accountability. Notably, trusts exhibiting a commitment-based response were more likely to have a history of higher performance (based on CQC ratings and interview data), greater organisational capacity, fewer infrastructure challenges, and favourable financial circumstances, which allowed them greater flexibility. These trusts did not, however, consistently show improvement against 7DS national audit data, perhaps reflecting their prioritisation of values-based change over external displays of compliance.

A more common response to these externally imposed standards was a compliance-based response. This response was displayed by trusts which were seen as having a hierarchical organisational culture, in which top-down directives were used to drive changes to service organisation and delivery. Organisations that displayed compliance-based approaches were generally able to demonstrate improvements against the standards for 7DS, but this type of approach prioritised meeting the standards over genuine engagement with goals of improving quality of care, and could result in dissatisfied and disenfranchised staff. For a minority of trusts, a severe lack of resources, and a pressing need to improve basic service quality were barriers to engaging with delivering against the 7DS agenda. These organisations at best, tried to demonstrate a level of compliance, and at worst, felt unable to respond to these additional demands given the other pressing challenges they were dealing with.

The link between organisational culture and improvement approach is not unexpected – the labels applied to cultural types relate to the ‘usual way of doing things round here’. The two trusts that were described as having a developmental culture, were also described by staff as having ways of doing things that were creative and flexible, and as working around common values and goals. This was seen as enabling them to resist the potential negative consequences of external performance management approaches to service-level improvement. Perhaps more importantly though, these two trusts had a history of high performance and a financial surplus, meaning that senior leaders were less concerned about having to demonstrate improvement. Previous success meant they felt they would be under less scrutiny and had more leeway to do things their own way, and had access to more resources to support improvement. As Burnett et al. observe, leaders are most concerned with delivering on the quality demands that affect the reputation or the funding of the hospital. When these targets are met, organisations can procure additional funds for quality improvement thus creating a virtuous circle leaving those without the ability to draw on these funds at an even greater disadvantage [3].

This study makes a novel contribution by taking an organisational behaviour perspective to understand responses at organisational level, to nationally imposed standards and targets. Regulatory and performance management approaches are likely to remain part of the strategy for improving healthcare in the future, particularly in centralised systems like the NHS in England. These approaches are likely to continue to be important in relation to the implementation of new policy, or to reduce unwanted variation and inconsistencies in service delivery. Our study suggests that some organisations, particularly those that are innovative, forward-looking, and well-resourced, may respond well to externally-imposed standards by using them as a springboard for commitment-based change. Perversely this may not be reflected in external audit data. For the majority of organisations, the imposition of standards and monitoring is likely to generate compliance-based approaches which may result in the desired changes on indicator measures, but may be limited in terms of embedding sustainable improvement. Failure to gain staff engagement can mean frontline staff may ‘working round’ top-down imposed standards while symbolically complying with them [20] For struggling organisations, additional requirements to meet performance standards may be unrealistic and introduce additional strain. These findings align with the findings of a systematic review of interventions to improve organisational performance across public sector organisations. The review identified: a tendency to define performance on the basis of external standards that focused on specific organisations as opposed to the system; a lack of adaptation of interventions to local context; and the potential for negative consequences of interventions when implemented in low performing organisations. The authors highlight the need to consider local context and the wider systems in which organisations operate in implementing improvement interventions, and the need to consider costs and negative consequences of interventions, particularly when applied to struggling organisations [21].

The NHS 7DS agenda was rolled out primarily through a national programme of standards and monitoring, employed with the aim of improving the delivery of weekend care to a standard level of performance across all local NHS trusts. This blanket approach had variable impact in the trusts we studied. Our findings suggest that efforts to implement large-scale change across organisations should be supported by a balance of different methods, including more attention to proactive, trust-specific, support for change [22]. Our findings underline the importance of contextually-sensitive approaches to driving improvement that reflect the extent to which organisations are under strain, and the resources available to them. For example, stretch targets might motivate high performing and well-funded organisations to innovate, while approaches such as reciprocal peer review [23], and targeted funding (e.g. for additional staff in new roles to enable change), might be more effective in helping struggling organisations improve. Our findings also suggest the need for external verification of improvement in response to standards, to provide a more nuanced assessment of levels of engagement with genuine improvement.

This study has limitations. The study was conducted in NHS trusts in England; research in other types of healthcare systems may identify different dynamics. We interviewed a small number of staff in each trust; perceptions of organisational culture were summarised to give an overall assessment, and we did not aim to capture subcultures within the organisation [5]. Senior staff perspectives are prominent in our findings as senior staff sitting at the apex of the organisation are in a unique position to define and shape local cultures through their influence over the choice of organisational strategy, allocation of resources as well as controlling systems for recruitment, retention and reward [24]. The sample within each trust was too small to provide a definitive assessment of organisational culture, but the use of the CVF during interviews allowed us to explore interviewee perceptions of how organisational culture impacted on implementation of the standards. The assessment of how well trusts were meeting the 7DS was based on published self-reported data on adherence to standards gathered as part of the national audit. We used the 7DS as a case study to investigate response of English NHS trusts to national standards, but acknowledge that evidence is lacking about whether in fact implementation of these standards can reduce mortality for patients admitted at weekends [25]. Our sample included only eight trusts, but these were selected to reflect a range with different specialist intensity at weekends, location and size. We were also able to recruit staff with different roles to provide a variety of perspectives within each organisation.

This qualitative study has generated hypothesis about relationships between culture, local context, and organisational response to national standards for service delivery. More research is needed to explore the relationships between these factors: a larger quantitative study of response to 7DS including assessments of organisational culture and local context, and independent assessments of improvement, would be of value.

## Conclusion

Our findings show how the introduction of national standards for service level improvement with performance monitoring can generate different types of response in different local settings. Externally-driven standards can be integrated into value-led organisational change strategies when organisations have a supportive culture and capacity for change; but may generate a tokenistic, compliance-based response when organisational culture and local context are less facilitative. When trusts are struggling, national quality standards may introduce additional strain. Approaches to driving improvement nationally need to be accompanied by resources and tailored support for improvement, taking into account organisational differences and local context.

## Supplementary Information

**Additional file 1.** Interview Guide. Interview topic guide used for interviews

**Additional file 2.** Coding frame

**Additional file 3.** Table participants by role

## Data Availability

The datasets used and/or analysed during the current study are available from the corresponding author on reasonable request.

## Abbreviations

7DS: 7-day service standards
CVF: Competing Values Framework
HiSLAC: High intensity specialist led care
NHS: National Health Service
