# Supplementary material for: A qualitative study of organisational response to national quality standards for 7-day services in English hospitals
Source: BMC Health Serv Res. 2021 Mar 6;21:205. doi: 10.1186/s12913-021-06213-w (PMC7937294; doi:10.1186/s12913-021-06213-w)
Supplement: Supplementary file 1 — Additional file 1. Interview Guide. Interview topic guide used for interviews [file 12913_2021_6213_MOESM1_ESM.docx]

# Additional file 1: Interview topic guide

*Introduce self, the HISLAC project. (If not already done) Remind participant to provide completed consent form. Provide indication of length of time they are required for. Check that interviewees consent to interview and use of data. Reiterate confidentiality. Aim of the interview is to understand how your particular trust operates with regards to the implementation of 7 day standards and 7 day services and to explore what the barriers and facilitators are to progressing them in your organisation.*

## Introduction

Could you tell me a bit about your role? What does it entail? What are you responsible for? How long have you been doing this?

## Board priorities/ 7 day services

How does the implementation of 7 day services fit in with other overall board priorities?

How is this organisation trying to implement 7 day services? What strategies are being used to drive these changes?

How are changes being communicated to others up and down the hierarchy?

Is there a dedicated person responsible for the oversight of implementing 7 day services?

- If so: What is their role?
- How long have they been in post?

## Organisational culture

*We are interested in your views about what sort of organisation you are and how your values and priorities might affect implementing change.*

Show diagram/Ask interviewee to look at diagram and ask interviewee to identify which of the 4 culture types best matches their organisation (hospital). Explain that all organisations are a mix of all four cultural types – we need to identify the dominant one or two.


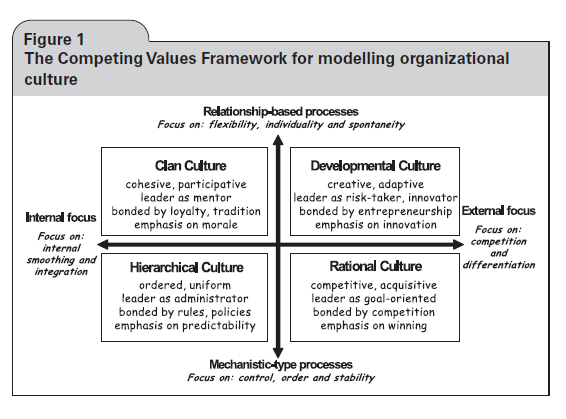
 J R Soc Med 2009: 102: 332–336.

How do you think the attributes/ values of the culture you’ve just highlighted affect the:

- Uptake of 7 day working practices – particularly specialist staffing at weekends?
- AND resistance to implementing 7 day working practices?

Why / in what way?

How has your organisational culture changed over the past 5 years? Has there been a transition from one cultural type to another (probe for which ones e.g. clan to rational or other).

Where would you like your organisation to be in the future? How do you think it will get there?

How do you think your organisation deals with change?

## 7 day services/standards

To what extent do you think you are currently meeting the 7 day service clinical standards?

- Probe on all 4 priority standards – 7 day provision of the following:
- Emergency admissions assessed by a suitable consultant within 14 hours from admission.
- Scheduled access to diagnostic services (eg ultrasound, CT, microbiology)
- Access to key consultant-directed interventions (e.g. critical care, interventional radiology, stroke thrombolysis)
- Consultant review for all high dependency patients TWICE DAILY; others at least ONCE EVERY 24 HOURS (unless determined it would not affect the patient’s care pathway)

**How** have you been able to meet these standards?

**Probe for** whether they are employing more specialists or using the existing workforce in a different way

If any that not currently meeting – why aren’t they?

What do you think needs to be done to improve?

We will also identify 3 or 4 key issues from the first round case studies from the observations and feed it back to the board to explore with them (e.g. specialist vs generalist acute team; who does patient review at weekends, and anything peculiar to the individual hospital e.g. GP with unique role in AMU).

## Delivery of specialist intensity

Has your trust been working to increase specialist staffing at the weekends?

- What is happening in this trust in relation to this? How is this going?
- Has the approach worked out as originally planned or have things changed? Why?
- What have been the positive intended outcomes? Any unintended outcomes?

What challenges or barriers are there for this trust in increasing specialist staffing at weekends? e.g. Financial constraints, certain groups’ resistance, other

What features of your organisation impact on your approach to increasing specialist staffing at the weekends? e.g. finances, mergers, organisational turbulence

How do factors outside your organisation affect increasing specialist staffing at weekends? e.g. Recruitment, funding, national policies
