# Supplementary material for: A qualitative study of organisational response to national quality standards for 7-day services in English hospitals
Source: BMC Health Serv Res. 2021 Mar 6;21:205. doi: 10.1186/s12913-021-06213-w (PMC7937294; doi:10.1186/s12913-021-06213-w)
Supplement: Supplementary file 2 — Additional file 2. Coding frame [file 12913_2021_6213_MOESM2_ESM.docx]

Additional file 2 Coding frame

| Name |
| --- |
| 001 INTERVIEWEE CHARACTERISTICS |
| 002 BOARD PRIORITIES |
| 003 DELIVERY OF SPECIALIST INTENSITY |
| 004 ORGANISATIONAL CULTURE |
| board perspectives |
| front lne views |
| 005 SEVEN DAY SERVICES |
| a) Which 7 day standards have been introduced |
| b) How have they gone about it |
| c) What role does culture play |
| d) What other factors impact on their ability to implement 7 day standards |
| 099 OTHER COMMENTS |
| General comments |
| Staff rotas |
| 100 WHAT HELPS IMPLEMENT CHANGE |
| Board views |
| front line views |
| 101 WHAT HINDERS IMPLEMENTING CHANGE |
| Board views |
| Front line views |
| interesting quotes |
