# Supplementary material for: A qualitative study of organisational response to national quality standards for 7-day services in English hospitals
Source: BMC Health Serv Res. 2021 Mar 6;21:205. doi: 10.1186/s12913-021-06213-w (PMC7937294; doi:10.1186/s12913-021-06213-w)
Supplement: Supplementary file 3 — Additional file 3. Table participants by role [file 12913_2021_6213_MOESM3_ESM.docx]

| **Trust** | **Med director** | **CEO** | **Finance Dir** | **Consultant** | **Nurse** | **Additional rota** | **Total** |
| --- | --- | --- | --- | --- | --- | --- | --- |
| 03 | 1 | 1 |  | 3 |  | 1 | **6** |
| 07 | 1 | 1 |  | 2 | 1 | 1 | **6** |
| **10** | 1 |  |  | 3 |  |  | **4** |
| 11 | 1 |  | 1 | 2 | 1 | 1 | **6** |
| 12 | 1 |  |  | 2 | 1 |  | **4** |
| 16 | 1 |  |  | 3 |  |  | **4** |
| 17 | 1 |  |  | 2 | 1 | 2 | **6** |
| 18 | 1 | 1 |  | 2 | 1 | 2 | **7** |
| **Total** | **8** | **3** | **1** | **19** | **5** | **7** | **43** |

Additional file 3 Breakdown of participants by role and by Trust
